# Glucocorticoid effects on the brain: from adaptive developmental plasticity to allostatic overload

**DOI:** 10.1242/jeb.246128

**Published:** 2024-03-07

**Authors:** Helen Eachus, Soojin Ryu

**Affiliations:** ^1^ Living Systems Institute & Department of Clinical and Biomedical Sciences, University of Exeter, Stocker Road, Exeter EX4 4QD, UK

**Keywords:** Allostasis, Stress, Neurogenesis, Neurodevelopment, Cortisol, Phenotypic plasticity

## Abstract

Exposure to stress during early life may alter the developmental trajectory of an animal by a mechanism known as adaptive plasticity. For example, to enhance reproductive success in an adverse environment, it is known that animals accelerate their growth during development. However, these short-term fitness benefits are often associated with reduced longevity, a phenomenon known as the growth rate–lifespan trade-off. In humans, early life stress exposure compromises health later in life and increases disease susceptibility. Glucocorticoids (GCs) are major stress hormones implicated in these processes. This Review discusses the evidence for GC-mediated adaptive plasticity in development, leading to allostatic overload in later life. We focus on GC-induced effects on brain structure and function, including neurogenesis; highlight the need for longitudinal studies; and discuss approaches to identify molecular mechanisms mediating GC-induced alteration of the brain developmental trajectory leading to adult dysfunctions. Further understanding of how stress and GC exposure can alter developmental trajectories at the molecular and cellular level is of critical importance to reduce the burden of mental and physical ill health across the life course.

## Teaser

**Summary:** Exposure to high levels of glucocorticoids can mediate adaptive plasticity during development, as well as allostatic overload in later life, especially in the context of the brain.

## Introduction

**Fig. 1.**
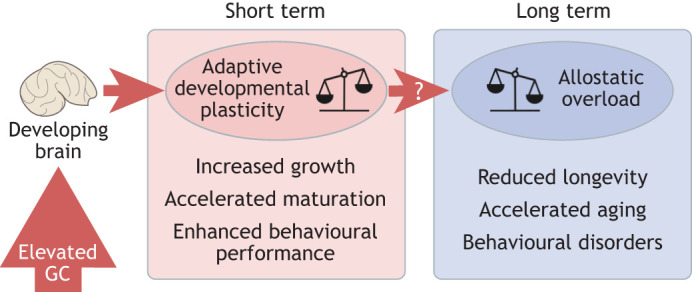
**Model proposing that elevated glucocorticoid (GC) mediates adaptive plasticity during development leading to allostatic overload in later life.** We suggest that in the short term, exposure to high levels of GCs can drive adaptive developmental plasticity, such as increased growth, accelerated maturation and enhanced behavioural performance. However, over time, chronic GC exposure leads to an accumulation of allostatic load, ultimately leading to a maladaptive state of allostatic overload. Allostatic overload may manifest via accelerated aging, reduced longevity and behavioural disorders in later life. Figure adapted from Eachus et al. (2023a preprint).

In vertebrates, the stress response is regulated by the well-conserved hypothalamo–pituitary–adrenal (HPA) axis, whose end effector is glucocorticoid hormone (GC). When an organism is exposed to stress, GCs exert pleiotropic effects on the body to restore homeostasis by activating adaptive mechanisms. This dynamic process is known as allostasis (McEwen and Wingfield, 2003). Over time, these allodynamic processes may promote adaptation, whereby an animal modifies its phenotype in response to a stimulus in a manner which increases its evolutionary fitness. This is known as adaptive plasticity. Indeed, exposure to high levels of GC is known to mediate numerous adaptive phenotypes which are likely to be beneficial when faced with adverse environmental conditions. One of the best examples of this is the accelerated growth of wild North American red squirrels, which correlates with exposure to high levels of maternal GCs (Dantzer et al., 2013, 2020). Other examples include behavioural alterations such as increased boldness in zebrafish larvae (Best et al., 2017); increased flight performance and wing maturation in fledgling sparrows (Chin et al., 2009); memory enhancement in rodents (Quirarte et al., 1997); and induction of spermiogenesis in zebrafish explants (Tovo-Neto et al., 2020). Ultimately, these traits may confer fitness advantages (Fig. 1).

**List of abbreviations**

AHN: adult hippocampal neurogenesis
DEG: differentially expressed gene
DG: dentate gyrus of the hippocampus
DMR: differentially methylated region
DNMT: DNA methyltransferase
ELS: early life stress
EMAL: energetic model of allostatic load
GC: glucocorticoid
GR: glucocorticoid receptor
GRE: glucocorticoid response element
HPA: hypothalamo–pituitary–adrenal axis
LTP: long-term potentiation
mPFC: medial prefrontal cortex
MR: mineralocorticoid receptor
NSPC: neural stem/progenitor cell

However, although exposure to stress might drive adaptive responses in the short term, long-term exposure to stressors can lead to accumulation of so-called allostatic load, and subsequently a maladaptive state of allostatic overload (McEwen and Wingfield, 2003). Examples of GC-induced allostatic overload can be observed across species. For example, in humans, exposure to elevated GC during early development, such as via early life stress (ELS) or antenatal GC treatment, is implicated in disease risk in later life (Lupien et al., 2009), especially of mental health disorders, and age-related disease. In animal studies, early life GC exposure is associated with reduced adult lifespan (Monaghan et al., 2012), HPA axis dysregulation (Casagrande et al., 2020; Haussmann et al., 2012), maladaptive behavioural phenotypes such as impaired fear extinction behaviour (Bingham et al., 2013), diminished behavioural flexibility (Reyes-Contreras and Taborsky, 2022) and impaired social competence (Reyes-Contreras et al., 2019). Such traits are often considered as biomarkers of behavioural disorders and accelerated aging (Pariante and Lightman, 2008; Kõks et al., 2016; Singewald and Holmes, 2019; Pietropaolo and Marsicano, 2022). The negative outcomes that typically manifest in later life can be considered as a trade-off for the short-term adaptive response to the stressor (Fig. 1). An example of this is the growth rate–lifespan trade-off, in which the cost of an earlier investment in growth and maturation is paid in later life in terms of reduced longevity (Lee et al., 2013).

Although a role for GCs in mediating adaptive plasticity and allostatic overload is established, the underlying mechanisms are not well understood (McEwen and Wingfield, 2003; McEwen and Liston, 2017; Lupien et al., 2009). In mammals, a surge in endogenous GC levels during foetal development provides an important developmental trigger for the maturation of many organ systems (Moisiadis and Matthews, 2014). As such, disruption of this critical signal, via exposure to excess GC, or altered timing of exposure potentially has the power to alter the developmental trajectory of the animal (Moisiadis and Matthews, 2014). Potentially the most at risk of the body's organs is the brain. During early life, the developing brain is plastic, in that its structure and function are highly susceptible to modification in response to internal and external cues. During early development, many species appear to go through a stress hypo-responsive period, during which the HPA axis is less responsive to external stressors (Schmidt, 2019). This period is thought to protect the developing brain from GC-induced modification or damage. As such, exposure to excess GC during this period has the power to significantly alter the developmental trajectory of the brain (McEwen and Gianaros, 2011).

In this Review, we will discuss the role of GCs in both adaptive developmental plasticity and allostatic overload in later life. Although some examples from human studies will be mentioned, the focus of this Review is on animal studies investigating underlying cellular and molecular mechanisms that are relevant to the brain. Our aim is to discuss exemplary studies that illustrate important concepts, and as such the Review is not meant to be an exhaustive coverage of the literature. We highlight the importance of longitudinal studies in connecting how GC-induced adaptive developmental plasticity might ultimately lead to allostatic overload in later life and suggest avenues for addressing the knowledge gap related to the role of GCs in conferring both adaptive and pathological phenotypes in the brain across the life course.

## GC-induced adaptive developmental plasticity

GCs are known to mediate both rapid non-genomic and delayed genomic effects that can modulate brain structure and function. Wide-ranging effects of GCs are mediated by GC receptors, glucocorticoid receptor (GR) and mineralocorticoid nuclear receptors (MRs), which upon GC binding can regulate transcription of target genes (Timmermans et al., 2019; Mifsud and Reul, 2018). In addition, rapid, non-genomic effects of membrane-bound GR and MRs have been reported (Groeneweg et al., 2012). Whilst GCs can bind both GR and MRs in the brain, where many cells express both types of receptors, GC has a high affinity for MRs. MR binding typically occurs at low levels of GC concentration, such as during resting state, whilst the lower affinity GR is typically occupied only under high GC concentration, such as under stress or at circadian peak (Reul et al., 1987). Whilst GR is expressed ubiquitously throughout the brain, MR expression is more restricted to limbic regions (Reul and de Kloet, 1985). In addition to GR and MRs, the role of other membrane receptors for GC that can mediate fast non-genomic effects of GC have been widely discussed (Panettieri et al., 2019). GC-induced adaptive plasticity has been studied in the context of the adult brain. For example, fear-conditioning-induced suppression of long-term potentiation (LTP) in the amygdala requires the GR and is thought to protect against excessive fear memory (Inoue et al., 2018). However, the role of GCs in adaptive developmental plasticity of the brain has not been widely studied. One example of structural plasticity that has been studied in the developing brain is dendritic spine remodelling (McEwen and Liston, 2017). New spines are formed in an ongoing process, and are mostly eliminated in a matter of days, whilst a subset will persist and form stable synapses. Although this process also occurs in the adult brain, levels of spine turnover are much higher in the developing brain, which undergoes rapid spinogenesis followed by protracted spine pruning (Liston and Gan, 2011). Spine remodelling is thought to be a critical mediator of learning and memory, because learning a motor skill induces brain-region-specific spine formation (Liston et al., 2013; Hayashi-Takagi et al., 2015), and spine shrinkage disrupts acquired motor learning (Hayashi-Takagi et al., 2015).

Work using transcranial time-lapse two-photon microscopy has shown that endogenous GC signalling is required for spine remodelling in the developing barrel cortex of adolescent mice and this can be enhanced by a single dose of GC treatment (Liston and Gan, 2011). In this context, developmental spine remodelling is primarily dependent on MR signalling (Liston and Gan, 2011). Further work has shown that learning-induced remodelling is mediated by circadian GC oscillations, whereby GC peaks facilitate formation of new spines and GC troughs stabilise the new spines, which enhances long-term memory retention (Liston et al., 2013). Here, spine formation was mediated at least in part by non-genomic activity of GR involving LIM Kinase 1 signalling; meanwhile, spine pruning was modulated by transcription-dependent signalling via MR activation. Together, these results support a role of GC-mediated spine remodelling involved in learning and memory during development.

Another example of GC-induced adaptive developmental plasticity is acceleration of brain maturation, including the effects of GC on neurogenesis and cell proliferation. Although GC exposure is known to accelerate lung maturation in the foetus, effects on other organs have been less well studied. In a mouse model, ELS accelerated neuronal maturation in the postnatal hippocampus (Bath et al., 2016). In ELS-exposed mice, the authors observed a precocious arrival of parvalbumin-expressing neurons, a cell cluster that typically develops late in postnatal hippocampal development. Parvalbumin-expressing hippocampal interneurons are essential for memory formation during early life (Miranda et al., 2022) and ELS accelerated the timed developmental suppression of contextual fear conditioning (Bath et al., 2016). There was also an earlier developmental switch in the expression ratio of *N*-methyl-d-aspartate (NMDA) receptor subunits, a marker of synaptic maturity, and an earlier rise in myelin basic protein (MBP) levels in the postnatal hippocampus (Bath et al., 2016), suggesting that ELS may promote an earlier neurodevelopmental switch from growth to maturation. A further study reported a GC-mediated acceleration of development in the cerebellum, whereby GC exposure was found to reduce cell proliferation but increase numbers of mature neurons in embryonic chicken granule neurons (Aden et al., 2011). Meanwhile, other studies have reported a GC-induced increase in cell proliferation. In a zebrafish model, maternal GC was found to increase cell proliferation in the pallium and the preoptic region and to upregulate expression of proneural gene *neurod4* in the embryonic brain (Best et al., 2017). Although the reported effects of developmental GC exposure on neurogenesis in the brain are mixed, with some reporting GC-induced reduction (Kanagawa et al., 2006), the studies discussed above support that exposure to elevated GC during development might, in some contexts, facilitate maturation and development of brain regions via altered neurogenesis.

It is proposed that accelerated neural maturation is part of a faster developmental strategy that would be advantageous in a high stress environment whereby an organism strives for earlier reproduction, as long-term survival may be uncertain (Callaghan and Tottenham, 2016). This is known as the stress acceleration hypothesis. However, over the long term, this developmental strategy is thought to impair plasticity, and in humans, ultimately increase vulnerability to psychiatric disorders in later life (Callaghan and Tottenham, 2016; Tooley et al., 2021). Because brain plasticity is known to reduce with age, it is thought that by accelerating maturation during early development, the window of heightened plasticity may close earlier (Tooley et al., 2021).

## GC-induced allostatic overload

In rodent models, one of the most consistent long-term effects of ELS on the brain is reduced adult hippocampal neurogenesis (AHN), which persists long after exposure to the stressor has ceased (Mirescu et al., 2004; Belnoue et al., 2013). The ELS-induced reduction in AHN includes a reduction in proliferation of progenitor cells and reduced production of new-born neurons (Mirescu et al., 2004; Aisa et al., 2009; Belnoue et al., 2013). Neurogenesis is a key mediator of brain plasticity, and the generation of new brain cells can provide an animal with adaptive capacity. Reduced AHN is often associated with defects in cognitive functions such as learning and memory in rodents (Lupien et al., 2009; Korosi et al., 2012). A loss of neurogenesis or depletion of the stem cell niche reduces the adaptive capacity of the brain (Bornstein et al., 2019; Surget and Belzung, 2022; Konefal et al., 2013). Indeed, an inability to respond adequately to a changing environment is a hallmark of aging (Matamales et al., 2016; Pettigrew and Martin, 2014; Rahner-Welsch et al., 1995). In adult mice, adrenalectomy can reduce the stress-induced reduction in AHN (Lehmann et al., 2013), and the reduction in cell proliferation in the hippocampus of ELS-exposed rats can be reversed by decreasing their cortisol level in adulthood (Mirescu et al., 2004). These studies support a role for GCs in modulating AHN during adulthood; however, despite the breadth of studies focusing on stress-induced effects on AHN in rodents, some of which are mentioned above, the effects of ELS or GC exposure on developmental neurogenesis or on the whole brain are relatively unknown. Also, evidence for a direct link between elevation of GC during early development and later life alteration of neurogenesis is lacking.

In addition to AHN, exposure to elevated GC is associated with structural and functional changes in the brain in human patients. This includes reduced white matter integrity of the whole brain (van der Meulen et al., 2022), global cerebral atrophy (Chen et al., 2020), and reduced grey matter volume of specific brain regions (Starkman et al., 1999). However, although most studies have focused on chronic or acute adult exposure to GC, rather than long-term effects of developmental GC, a report in humans found that foetal GC exposure was associated with cortical thinning in children (Davis et al., 2013), suggesting that exposure to elevated GC even for a short duration during development might have long-lasting impacts on brain structure. Further, a study of adolescents who were exposed to antenatal GC treatment found reduced functional connectivity in a brain network involving sub-cortical, cerebellar and frontal nodes (Magalhães et al., 2023 preprint). Meanwhile, in guinea pigs, LTP was depressed in the juvenile hippocampus following acute GC treatment, but acute GC treatment had no effect on females that were previously exposed to prenatal GCs, suggesting that prenatal GC can lead to long-term alteration of brain function under stress (Setiawan et al., 2007). These studies support a role of GCs in mediating long-term or delayed effects on structure and function of the brain *in vivo*.

## Where is the tipping point?

Although some evidence can be found for a role of GCs in mediating both short-term adaptive plasticity during brain development and long-term or delayed effects reminiscent of a maladaptive state of allostatic overload in the brain, there is a lack of connection between these two processes (Fig. 1). Concepts including the growth rate–lifespan trade-off and early life programming of adult disease make connections between early life and later life phenotypes; however, empirical support for such theories requires long-term studies that analyse the trajectory of brain development across the life course.

A small number of studies have analysed hippocampal neurogenesis across the life course, following developmental exposure to GC. In one study, pregnant mice were exposed to a single dose of GC, and hippocampal neurogenesis and volume were monitored in the offspring across embryogenesis, postnatal development and into adulthood (Noorlander et al., 2014). The authors observed some temporary effects, including a temporary increase in embryonic apoptosis, increased number of dentate gyrus (DG) neurons during the postnatal period, and reduced body mass and total hippocampal volume within the postnatal period. Meanwhile, cell proliferation was initially reduced in the embryonic DG, followed by an increase during the postnatal period and a subsequent reduction during adulthood (Noorlander et al., 2014), suggesting life-long and temporally dynamic effects of GC on neurogenesis, with potential implication for hippocampal-related cognitive functions. Further studies using a variety of animal models are required to identify the developmental dynamics of neurogenesis across the life course following GC exposure.

Our recent work analysed the effects of developmental GC exposure on neurogenesis in an optogenetic zebrafish model (Eachus et al., 2023a preprint). We observed a striking brain-region-specific effect of GC on cell proliferation that was restricted to the developing hypothalamus. In GC-exposed fish, hypothalamic cell proliferation was initially increased, and was primarily restricted to a population of *rx3*-expressing radial glia that reside within the proliferative ventricular region. The mammalian orthologue of *rx3*, *Rax*, is expressed in hypothalamic tanycytes, an intriguing cell population that undergoes adult neurogenesis (Goodman and Hajihosseini, 2015) and is known to be diet (Lee et al., 2012) and stress responsive (Bielefeld et al., 2021). *rx3/Rax* plays a role in hypothalamic development in fish and rodents (Muthu et al., 2016; De Souza and Placzek, 2021); however, whether the broader properties of hypothalamic tanycytes are conserved in fish is currently unknown. GC appears to directly regulate *rx3* gene expression, as we could show that GR binds to glucocorticoid responsive elements (GRE) within the promotor of *rx3* (Eachus et al., 2023a preprint). In GC-exposed animals, excess proliferation coincided with an increase in neuronal precursor cells, an overall increase in hypothalamic volume, and early emergence of feeding, a hypothalamus-associated behaviour. Interestingly, this GC-induced precocious hypothalamic development could not be sustained, and in later development we observed a rapid decline in hypothalamic neurogenesis (Eachus et al., 2023a preprint). Under chronic high GC, growth of the hypothalamus slowed down, proliferative radial glia were lost, and differentiated hypothalamic neurons known to regulate feeding were reduced in number, correlating with a reduction in feeding. Ultimately, we observed general physical decline in fish exposed to developmental GC, including impaired growth and fertility, and reduced longevity (Eachus et al., 2023a preprint). These data provide cellular and molecular level insight that supports a model in which developmental GC exposure drives short-term adaptive plasticity but ultimately leads to allostatic overload in a developing brain and identifies a developmental time window that serves as a tipping point.

## Mechanisms underlying GC-mediated plasticity leading to allostatic overload

### Energetic cost of allostasis

One of the well-known roles of GCs in the body is mobilisation of energy resources and GC is known to positively correlate with organismal energy expenditure (Haase et al., 2016). Chronic GC exposure can trigger a state of cellular hypermetabolism (Bobba-Alves et al., 2023). Indeed, the allostatic process of GC-induced adaptive plasticity likely generates additional energetic burden, known as the energetic model of allostatic load (EMAL) (Bobba-Alves et al., 2022). This model proposes that a transition from adaptive plasticity to allostatic overload may occur when the added energetic cost of allostasis competes with longevity-promoting growth, maintenance and repair, leading to progressive wear-and-tear on the body (Bobba-Alves et al., 2022). Further, the model supports that systems that require continuous renewal to maintain function, such as brain regions with high levels of neurogenesis (e.g. the hippocampus and hypothalamus), are especially vulnerable to the above-mentioned trade-off. This vulnerability is likely exacerbated during early development, when energy costs and rates of neurogenesis are highest.

Empirical support for the EMAL model includes longitudinal studies of the effect of chronic GC exposure on human fibroblast cells (e.g. Bobba-Alves et al., 2023). In this study, chronic GC caused persistent hypermetabolism across the treatment duration, and led to a progressive increase in cell death and reduction in cell volume. Further, chronic GC accelerated the rate of telomere shortening and the rate of epigenetic aging across the cellular lifespan, linking GC-induced energetic demand with accelerated aging phenotypes. Interestingly, although chronic GC slowed down the rate of cell division, it also increased the energetic cost per round of cell division. Indeed, cell division is an energetically costly process (Salazar-Roa and Malumbres, 2017).

As such, it is plausible that in our aforementioned zebrafish model of adaptive plasticity leading to allostatic overload, the initial increase in cell proliferation induced by GC generates a significant energic burden, which cannot be maintained and potentially comes at a cost to energetic investment in growth, maintenance, and repair over the long term.

### Dose- and context-dependent effects of GC signalling factors

One of the well-documented aspects of GC-induced effects on the brain is the inverted U-shaped dose–response relationship (Joëls, 2006). This relationship, which is known to be brain-region-specific, describes how lower or higher doses of GC might have opposite effects to each other on various aspects of brain structure and function. In many cases, low doses of GC typically lead to positive outcomes, whereas high doses appear to produce detrimental effects on the brain. The inverted U-shaped response pattern has been related to effects of GC on many different aspects of brain structure and function, including neurogenesis and behaviour. Spatial learning and memory in rodents (Quirarte et al., 1997), innate behaviours in zebrafish (Ryu and De Marco, 2017), long-term potentiation (Diamond et al., 1992) as well as mitochondrial functions (Du et al., 2009) are only some of the examples that have been shown to exhibit inverted U-shaped responses correlated with dose of GC exposure. In the brain, it is thought that the U-shaped response to GCs is mediated at least is part by the dual action of the GR and MR receptor systems, which act in opposing directions, as well as by the expression of receptor variants in specific cell types (Joëls, 2006). In human hippocampal progenitor cells, exposure to a low concentration of GC led to increased cell proliferation, but decreased neurogenesis and increased astrogliogenesis (Anacker et al., 2013). Meanwhile, a higher concentration of GC decreased cell proliferation and neuronal differentiation, without affecting astrogliogenesis. These effects were dependent on MR and GR activation, respectively. Recent papers highlight that the role of GR and MR in the stress response can be complex and context-dependent (Koning et al., 2019; Daskalakis et al., 2022). Indeed, MR and GR bind at overlapping and distinct loci in the rodent hippocampus and are associated with distinct transcription factor binding motif landscapes (Mifsud et al., 2021).

Further, acute and chronic exposure to GC is known to produce contrasting effects on the brain. Although in the short term, GC signalling via GR produces adaptive homeostatic responses in the brain and body, chronic exposure to GC can lead to so-called GC resistance. GC resistance can occur when expression of GR is reduced, typically via altered methylation of the promotor region of the GR encoding gene *NR3C1* (*nuclear receptor subfamily 3 group C member 1*), leading to reduced GR protein availability and subsequent impaired GR signalling and lack of negative feedback to the HPA axis (Liu and Nusslock, 2018b; Eachus and Cunliffe, 2018). Early life exposure to GC is known to reduce GR levels in the hippocampus, hypothalamus and prefrontal cortex in adult rats (Bingham et al., 2013). Indeed, signalling via GR, rather than MR, is thought to be responsible for GC-induced reductions in neurogenesis, and it has been proposed that the level of GR activation exhibits a U-shaped relationship relative to neurogenesis (Saaltink and Vreugdenhil, 2014). In this model, acute or controllable stress, such as physical activity and environmental enrichment, drive ‘proper’ activation of GR, leading to increased neurogenesis, whereas chronic or uncontrollable stress might lead to high GR activity, inhibiting neurogenesis. In support of the latter, in cultured rat embryonic neural stem/progenitor cells (NSPCs), GC exposure induced a reduction of cell proliferation by inhibiting cell cycle progression via reduced expression of cyclin D1, and these effects were blocked by administration of a GR antagonist (Sundberg et al., 2006). However, direct evidence for how GR mediates contrasting context-dependent effects on neurogenesis is lacking. Interestingly, work on meta-plasticity in the amygdala also identifies different roles for the GC receptor in mediating brain function over different durations. *In vitro* slices of mouse amygdala exhibit a rapid enhancement of glutamatergic transmission in response to acute GC exposure, which was mediated by MR (Karst et al., 2010). Interestingly, this effect is long-lasting, and affects the response to a second hit of GC, which inhibits glutamatergic transmission, and is mediated by GR. In this context, the amygdala exhibited a switch in neurotransmission following repeated exposure to GC via differential activity of GC receptors.

The role of GCs in mediating AHN is complex. Although exposure to ELS or GC is robustly linked with reduced AHN in rodents, factors that may increase endogenous GC levels, such as environmental enrichment and exercise, can actually increase rates of AHN (Saaltink and Vreugdenhil, 2014). This seemingly conflicting phenomenon may be linked with the exact GC level induced by the treatment, as well as receptor activity. Exercise has been shown to stimulate adult neurogenesis in a variety of contexts in rodents (Trinchero et al., 2019; Liu and Nusslock, 2018a); however, exercise may also elevate endogenous cortisol levels (Hill et al., 2008). In a mouse study, animals that were subjected to chronic moderate exercise exhibited increased basal cortisol levels, and increased cell proliferation, differentiation, neuronal survival and migration, whilst also exhibiting an improvement in spatial pattern separation (So et al., 2017). However, animals subjected to chronic intense exercise had higher GC levels relative to controls, and although they did have increased neuronal differentiation and migration, no differences were observed in proliferation, survival or learning behaviour (So et al., 2017). Interestingly, these effects correlated with an increase in BDNF level in modest exercisers, but not in intense exercisers. These results suggest an intensity-dependent effect of exercise on neurogenesis, and suggest that moderate exercise, inducing a moderate increase in cortisol level, may represent a ‘sweet spot’. Saaltink and Vreugdenhil (2014) proposed a model in which exercise represents a form of controllable stress, promoting ‘proper’ GR activity and stimulating neurogenesis via factors such as BDNF. The authors argue that the exact GR expression level directly regulates the excitation–inhibition balance, which is critical for normal neurogenesis (Saaltink and Vreugdenhil, 2014).

Another player involved in GC signalling in the developing brain is 11β-hydroxysteroid dehydrogenase 2 (11β-HSD2), which catalyses the conversion of cortisol and corticosterone into inert cortisone and 11-dehydrocorticosterone (Chapman et al., 2013). In mammals, the *11β-HSD2* gene is expressed at high levels in the placenta and the foetal brain (Brown et al., 1996; Chapman et al., 2013), where it is thought that *11β-HSD2* functions to protect the developing animal from potentially deleterious effects of exposure to high GC. In contrast, in the adult brain, expression of *11β-HSD2* is very low and is expressed in a few brain regions including the nucleus tractus solitarus, where it is thought to confer aldosterone specificity to the MR by inactivating GCs (Wyrwoll et al., 2011). In support of the role of *11β-HSD2* in protecting against early life programming of adult disease by GCs, deletion of *11β-HSD2* in the foetal brain leads to depression-like behaviours and cognitive dysfunction in adult mice (Wyrwoll et al., 2015). Further, in the developing mouse brain, loss of *11β-HSD2* leads to reduced growth of the cerebellum and delay of neurodevelopmental landmarks such as negative geotaxis and eye opening (Holmes et al., 2006).

### GC-inducible stem cells

Recently, the concept of GC-inducible stem cells proposed that effects of stress or GC on NSPCs in young individuals may affect their renewal potential in the long-term, predisposing to adult disease (Bornstein et al., 2019). This concept is fitting with the idea discussed here, in that developmental GC exposure may alter neurogenesis or cell fate during early life, thus altering the developmental trajectory of the brain, ultimately leading to a pathological state reminiscent of allostatic overload. Previous work has indicated that proliferation of NSPCs may be limited to a finite number of cell division cycles before they differentiate into astrocytes, ultimately leading to a depletion of the NSPC pool (Encinas et al., 2011). In support of this, a previous study demonstrated that in the mouse, ELS led to an initial increase in hippocampal cell proliferation during early postnatal life, which was followed by a reduction in hippocampal NSPCs in adulthood (Youssef et al., 2019), where the authors reasoned that enhanced cell proliferation in early life had likely depleted the stem cell pool over time. Another study observed premature differentiation of medial prefrontal cortex (mPFC) oligodendrocytes following ELS in mice, leading to depletion of the oligodendrocyte progenitor cell pool (Teissier et al., 2020).

It is likely that to some extent NSPCs are regulated directly by GC via GR. The majority of quiescent and proliferating NSPCs express GR, as do mature neurons; meanwhile, GR is downregulated during differentiation, suggesting that GC likely has dynamic effects on different stages of neurogenesis and different cell types (Egeland et al., 2015). Further, the circadian and ultradian rhythmicity of GC oscillations is known to influence GC effects on NSPCs and neurogenesis (Fitzsimons et al., 2016). A recent study demonstrated a role for GCs in NSPC activation (Schouten et al., 2020). Circadian GC oscillations were shown to control cell cycle progression *in vitro* and *in vivo* in the mouse brain and to induce specific DNA methylation profiles *in vitro*, some of which were long lasting and related to WNT signalling. In the aging mouse brain, it was found that endogenous GC oscillations maintain hippocampal NSPCs in a quiescent state, and this was mediated by GR. Indeed, the proportion of this population of hippocampal NSPCs that express GR was found to increase with age, when GC oscillations also have increased magnitude, suggesting that NSPCs in the aging hippocampus may be especially sensitive to GC and stress. In addition to direct activation of NSPCs via GR, GC might also cause indirect activation. In rats, acute stress induced proliferation of NSPCs in the adult hippocampus, and this was mediated by GC effects on astrocytes that secreted FGF2 (Kirby et al., 2013). Further, ELS-induced effects on mPFC oligodendrocyte differentiation were found to be mediated by neuronal activity (Teissier et al., 2020).

### GC-induced senescence and stemness exhaustion

In the context of neurogenesis, a potential mediator of a transition from a GC-induced increase in cell proliferation to a subsequent reduction of cell proliferation is replicative senescence. Replicative senescence can occur following excess cell proliferation, where telomere length has reached a critical lower limit following a certain number of cell divisions, imposing a functional limit of cell replication. Cells in a senescent state incur an irreversible cell cycle arrest, yet remain viable, have alterations in metabolic activity and undergo dramatic changes in gene expression (Kumari and Jat, 2021). Exposure to GC is known to induce senescence *in vitro*. In liver progenitor cells, GC exposure induced cell proliferation in a subset of formerly quiescent progenitors via upregulation of galectin-3 (Yang et al., 2020). This GC-induced proliferation ultimately led to long-term replicative senescence and so-called stemness exhaustion. Meanwhile, GC exposure can induce a reversible cell dormancy state in a lung cancer model, mediated by the known GR target gene *CDNK1C* (*cyclin-dependent kinase inhibitor 1C*) (Prekovic et al., 2021), suggesting that GC exposure can regulate cell proliferation and cell state via GR-mediated regulation of the cell cycle. In this pathological context, the GC-induced reduction in proliferation likely represents an adaptive and/or protective mechanism. Further, it was recently demonstrated that circadian GC signalling in the hippocampus maintains NSPC quiescence in the context of the aging brain (Schouten et al., 2020). These studies demonstrate a role for GCs in mediating cell dormancy, a state that is likely required for health in some contexts and may be lost with aging or in disease. However, the role of GCs in mediating cell state during brain development may differ and is currently unclear.

In addition to senescence, GC exposure may lead to other features associated with cellular aging, reminiscent of allostatic overload. In human fibroblast cells, chronic GC exposure led to altered extracellular cytokines and cell-free DNA, increased mitochondrial DNA instability, telomere shortening and reduced cellular lifespan (Bobba-Alves et al., 2023). Interestingly, in that study, replicative senescence was reached earlier following GC treatment, but this did not result from an increase in the number of cell divisions. In fact, GC slowed down the rate of population doubling. Thus, the GC-induced telomere shortening observed was not a result of increased cell proliferation. Other studies have also reported an increase in cellular senescence, alongside a long-lasting reduction of cell proliferation (Bose et al., 2010). An alternative mediator of the GC-induced senescence is an increase in reactive oxygen species, leading to telomere attrition. Exposure to developmental GC is known to generate oxidative stress (Haussmann et al., 2012), and the brain is especially susceptible to this (Costantini et al., 2011), whilst oxidative stress is a well-known cause of DNA damage and telomere attrition (Metcalfe and Olsson, 2022).

### GC-induced epigenetic mechanisms affecting the developmental trajectory

The epigenome is a potential direct mechanistic link between early life experience and later life outcomes. Indeed, studies in humans and animal models support that ELS can drive stable changes to the epigenome, often linked with altered gene expression and adverse behavioural outcomes in later life (Jawahar et al., 2015; Provençal and Binder, 2015; Torres-Berrío et al., 2019). Many studies have demonstrated that GCs can shape the epigenome through mechanisms including demethylation at or near GREs (Wiechmann et al., 2019), histone modifications (Ito et al., 2000), regulation of miRNAs (Dwivedi et al., 2015), and chromatin remodelling (Vockley et al., 2016). The large amount of literature on epigenetic changes induced by GCs is covered by recent reviews (Mourtzi et al., 2021; Zannas and Chrousos, 2017; Gray et al., 2017) and is beyond the scope of this Review. Instead, below we discuss a few exemplary studies of GC-induced epigenetic mechanisms identified in the developing brain with long-lasting effects.

Studies have analysed the effects of GC exposure on genome-wide DNA methylation patterns, observing profound changes both *in vitro* and *in vivo* in the rodent brain (e.g. Bose et al., 2015); however, the function of these changes is often unclear. It is known that there are organ-specific developmental trajectories of DNA methylation, and prenatal exposure of guinea pigs to synthetic GC was shown to substantially modify these trajectories, including long-lasting changes to global DNA methylation levels in various organs that persist until adulthood or even the next generation (Crudo et al., 2012). Comparative analysis of GC-induced changes to DNA methylation patterns in the hippocampus and peripheral blood samples supports the incidence of both tissue-specific and common methylation signatures of GC exposure across the genome in rodents (Sasaki et al., 2021; Seifuddin et al., 2017).

One of the classic examples of epigenetic programming of early life experience is altered methylation of the GR-encoding gene *NR3C1*, whereby ELS drives hypermethylation of the regulatory region, attenuating GR expression and subsequent function (Liu and Nusslock, 2018b). In the guinea pig hippocampus, prenatal exposure to GC was shown to have contrasting short- and long-term effects on gene expression, GR–DNA binding and DNA methylation (Crudo et al., 2013). GC is also implicated in altered methylation of the *FKBP5* (*FK506 binding protein 5*) gene. FKBP5 is a co-chaperone of GR and its binding to the GR complex reduces the affinity of GCs to GR and delays translocation of GR into the nucleus (Zannas et al., 2016). Importantly, in humans, demethylation at specific GREs within the *FKBP5* gene is associated with long-term dysregulation of the stress hormone system and increased risk of developing stress-related psychiatric disorders in adulthood (Klengel et al., 2013). In the amygdala of GC-treated mice, methylation at a GRE within the *FKBP5* gene and subsequent expression of *FKBP5* were altered in a dose- and time-dependent manner and correlated with altered fear extinction behaviour (Sawamura et al., 2016). In that study, a lower dose of GC led to a reduction in *FKBP5* expression and a trend towards an increase in methylation, meanwhile a higher GC dose led to a reduction in methylation and an increase in *FKBP5* expression in the amygdala, 2 h after fear extinction learning (Sawamura et al., 2016). Meanwhile in the mouse, chronic GC exposure led to decreased DNA methylation at specific CpGs and increased expression of *FKBP5* in the hippocampus and hypothalamus (Lee et al., 2010). These effects correlated with altered expression of HPA axis genes, including reduced expression of *NR3C1*, and with anxiety-like behaviour in GC-exposed mice. These studies support that developmental exposure to GC might exert changes to brain development and function via altered methylation of *NR3C1* and *FKBP5*.

In the zebrafish, loss of GR function leads to widespread alterations to the adult brain methylome and transcriptome (Eachus et al., 2023b). This includes a DMR within the *fkbp5* gene, which exhibits hypermethylation and reduced expression in brains of GR mutants. Genes associated with GR-sensitive DMRs were linked to biological processes including GC response and neurogenesis; meanwhile, GR-sensitive DEGs were strongly associated with chaperone-mediated protein folding, the regulation of circadian rhythm, and the regulation of metabolism. GR mutant zebrafish exhibit striking behavioural abnormalities, including anxiety-like behaviours (Eachus et al., 2023b; Ziv et al., 2013). Interestingly, a subset of GR-sensitive DEGs in the zebrafish brain, including *bdnf*, are associated with behaviour, and some are implicated in depression and anxiety in humans (Eachus et al., 2023b). These data identify novel molecular mechanisms through which GR might modulate behaviour and GC signalling in the brain.

Prenatal exposure to GC in guinea pigs was shown to alter methylation of genes associated with brain development in the hippocampus of juveniles (Sasaki et al., 2021), indicating that early life exposure to GC might lead to long lasting effects on the brain developmental trajectory via the methylome. Similarly, our results in zebrafish indicate that developmental GC exposure leads to long-lasting changes in the level of some epigenetic modulators in the brain (Choi et al., 2023 preprint). In GC-exposed zebrafish, the expression level of several DNA methyltransferases (DNMTs) are altered, including *dnmt3aa* and *dnmt3bb.*3 (orthologous to DNMT3a and −3b) (Okano et al., 1999), which can alter the global DNA methylation landscape across the life course (Choi et al., 2023 preprint). Although *dnmt3bb.*3 exhibited a transient upregulation in GC-exposed fish during early life, *dnmt3aa* exhibited long-lasting changes in expression level in adulthood, long after GC exposure had ceased.

Altered methylation of specific genes involved in brain development has been analysed in response to ELS or GC, such as *BDNF*, which plays multiple roles in neurodevelopment and is implicated in psychiatric disorders (Boulle et al., 2012). In mice exposed to prenatal stress, epigenetic regulation of *BDNF* was implicated in the development of depressive- and anxiety-like behavioural phenotypes (Zheng et al., 2016). Methylation at specific promotors on *BDNF* were increased following prenatal stress and associated with reduced *BDNF* expression in the hippocampus.

A recent study investigated the role of GCs in modulating DNA methylation in the context of neurogenesis. GC treatment of a hippocampal progenitor cell line during proliferation and differentiation identified both short-term and long-term effects on the transcriptome and methylome (Provençal et al., 2020). The differentially expressed genes (DEGs) and differentially methylated regions (DMRs) identified after GC treatment during the cell proliferation phase treatment were mostly short-term, whilst only some were long-lasting. Interestingly, more of the methylation changes were long-lasting than expression changes. Meanwhile, some DMRs had methylation trajectories which changed across the differentiation time window, suggesting a mechanism by which GC might exert dynamic effects on neurogenesis across development. Interestingly, a subset of DMRs was responsive to a subsequent acute GC challenge, indicating that early GC exposure has changed the setpoint for subsequent stress responses. Across neurogenesis, the DEGs and DMRs were enriched in pathways associated with neurogenesis and regulation of transcription, but interestingly, long-lasting DMRs were associated with a specific group of pathways including axon development, actin filament organisation and small guanosine triphosphate phosphohydrolase (GTPase)-mediated signal transduction (Provençal et al., 2020).

The epigenome may also play a role in GC-mediated accelerated aging, with epigenetic effects of GC exposure accumulating across the lifespan that are linked with age-related disease (Zannas and Chrousos, 2017). In a human cohort study, cumulative lifetime stress was found to predict DNA methylation patterns at so-called epigenetic clock genes (Zannas et al., 2015), where methylation patterns are able to predict chronological age (Horvath, 2013). Interestingly, an acute exposure to GC was sufficient to induce altered methylation and transcription of a significant number of these genes, and these genes showed an enriched association with age-related diseases (Zannas et al., 2015). Methylation of *FKBP5* is also known to decrease with aging in humans, and in a model of replicative senescence, the *in vitro* aging-induced decline in *FKBP5* methylation was exacerbated by GC exposure and the subsequently increased *FKBP5* expression was associated with inflammation and myocardial infarction (Zannas et al., 2019). These studies implicate GC in mediating age-related diseases via epigenetic mechanisms.

## Outlook and future perspectives

This Review discussed the growing body of evidence that links GC-mediated adaptive plasticity during development with allostatic overload in later life. The developmental origin of adult disease is a well-established phenomenon in humans, and as a main stress hormone shaping an organism's allostasis, GC is likely to be a key player in defining adult fitness as well as dysfunction. Although molecular- and cellular-level understanding of how GC-induced adaptive plasticity during development is rapidly emerging, we currently lack both conceptual and experimental insights into how this process becomes maladaptive. Some of the key basic questions regarding this process are currently unanswered. When does the GC-mediated accelerated growth stop and what triggers it? How do cells determine the tipping point between accelerated growth and stalled development? What are the differences among distinct cell types and brain regions in response to GC and how are these different responses coordinated to produce adaptive fitness in an organism?

A key strategy in answering these questions, we argue, is the need for longitudinal studies that examine the effect of GC across the life course of an animal. Experimental animal models such as our optogenetic transgenic zebrafish model, which allows elevation of endogenous GC levels at will, is ideally suited to serve this purpose. As zebrafish develop externally and are easy to raise in large numbers, long-term longitudinal studies are feasible. As a function of specific GC exposure, changes in the brain across the life course can be studied comprehensively at the molecular and cellular level using rapidly advancing multiomic and high-resolution imaging techniques, and linked to behavioural and functional consequences in adulthood.

A particularly fruitful area for near-term advance is determining how GC affects the development of stem cells and progenitor cells. Determining how GC exposure alters the molecular and cellular developmental trajectory of these cells using single cell techniques including lineage tracing and sequencing will reveal the underlying mechanism by which GC changes cell fate across the life course. Identified molecular changes can be manipulated in experimental animal models to ameliorate the effects of GC-induced allostatic overload.

Although most studies to date have used heterogeneous brain tissues to reveal GC-induced epigenetic changes, cell-type-specific epigenetic modifications will be most informative in linking GC exposure with specific phenotypic alterations (Rahman and McGowan, 2022). The advances in single cell sequencing technologies offer enormous opportunity to achieve this. Future studies that combine the study of cell-type-specific epigenetic modifications with temporal analysis will be key to revealing how and whether epigenetic modifications in specific cell populations mediate GC-induced alterations in gene expression and behaviour that manifest across different time scales.

The enormous impact of sex differences is becoming more and more apparent. It is known that there are sex differences in susceptibility to stress-related disorders and GC programming (Carpenter et al., 2017), as well as in stress-induced structural remodelling in the brain (Gray et al., 2017) and genome-wide transcriptional and epigenetic signatures of ELS (Parel and Peña, 2022). Further, sex differences in stress regulation are also linked with the hypothalamo–pituitary–gonadal axis, and thus differences may emerge during periods of dynamic hormonal fluctuations, such as during adolescence, as well as in aging (Bale and Epperson, 2015). As such, analysis of sex as a factor in determining the effects of GCs on the brain and behaviour in both human studies and animal models, especially longitudinal studies, will add value to this research field and may aid the development of more targeted therapeutics for stress-related disorders.

In conclusion, recent cellular and molecular advances offer important clues about the mechanisms underlying GC-induced adaptive plasticity leading to allostatic overload. Time is ripe for comprehensive longitudinal life course studies in experimental animals to identify and validate causal mechanisms underlying GC-induced allostatic overload. Such knowledge holds great promise for improving understanding of a variety of human diseases with developmental origin.

## References

[JEB246128C1] Aden, P., Paulsen, R. E., Maehlen, J., Loberg, E. M., Goverud, I. L., Liestol, K. and Lomo, J. (2011). Glucocorticoids dexamethasone and hydrocortisone inhibit proliferation and accelerate maturation of chicken cerebellar granule neurons. *Brain Res.* 1418, 32-41. 10.1016/j.brainres.2011.08.053 21925649

[JEB246128C2] Aisa, B., Elizalde, N., Tordera, R., Lasheras, B., Del Rio, J. and Ramirez, M. J. (2009). Effects of neonatal stress on markers of synaptic plasticity in the hippocampus: implications for spatial memory. *Hippocampus* 19, 1222-1231. 10.1002/hipo.20586 19309038

[JEB246128C3] Anacker, C., Cattaneo, A., Luoni, A., Musaelyan, K., Zunszain, P. A., Milanesi, E., Rybka, J., Berry, A., Cirulli, F., Thuret, S. et al. (2013). Glucocorticoid-related molecular signaling pathways regulating hippocampal neurogenesis. *Neuropsychopharmacology* 38, 872-883. 10.1038/npp.2012.253 23303060 PMC3672002

[JEB246128C4] Bale, T. L. and Epperson, C. N. (2015). Sex differences and stress across the lifespan. *Nat. Neurosci.* 18, 1413-1420. 10.1038/nn.4112 26404716 PMC4620712

[JEB246128C5] Bath, K. G., Manzano-Nieves, G. and Goodwill, H. (2016). Early life stress accelerates behavioral and neural maturation of the hippocampus in male mice. *Horm. Behav.* 82, 64-71. 10.1016/j.yhbeh.2016.04.010 27155103 PMC5308418

[JEB246128C6] Belnoue, L., Grosjean, N., Ladeveze, E., Abrous, D. N. and Koehl, M. (2013). Prenatal stress inhibits hippocampal neurogenesis but spares olfactory bulb neurogenesis. *PLoS One* 8, e72972. 10.1371/journal.pone.0072972 24009723 PMC3756947

[JEB246128C7] Best, C., Kurrasch, D. M. and Vijayan, M. M. (2017). Maternal cortisol stimulates neurogenesis and affects larval behaviour in zebrafish. *Sci. Rep.* 7, 40905. 10.1038/srep40905 28098234 PMC5241638

[JEB246128C8] Bielefeld, P., Abbink, M. R., Davidson, A. R., Reijner, N., Abiega, O., Lucassen, P. J., Korosi, A. and Fitzsimons, C. P. (2021). Early life stress decreases cell proliferation and the number of putative adult neural stem cells in the adult hypothalamus. *Stress* 24, 189-195. 10.1080/10253890.2021.1879787 33494651

[JEB246128C9] Bingham, B. C., Sheela Rani, C. S., Frazer, A., Strong, R. and Morilak, D. A. (2013). Exogenous prenatal corticosterone exposure mimics the effects of prenatal stress on adult brain stress response systems and fear extinction behavior. *Psychoneuroendocrinology* 38, 2746-2757. 10.1016/j.psyneuen.2013.07.003 23937971

[JEB246128C10] Bobba-Alves, N., Juster, R. P. and Picard, M. (2022). The energetic cost of allostasis and allostatic load. *Psychoneuroendocrinology* 146, 105951. 10.1016/j.psyneuen.2022.105951 36302295 PMC10082134

[JEB246128C11] Bobba-Alves, N., Sturm, G., Lin, J., Ware, S. A., Karan, K. R., Monzel, A. S., Bris, C., Procaccio, V., Lenaers, G., Higgins-Chen, A. et al. (2023). Cellular allostatic load is linked to increased energy expenditure and accelerated biological aging. *Psychoneuroendocrinology* 155, 106322. 10.1016/j.psyneuen.2023.106322 37423094 PMC10528419

[JEB246128C12] Bornstein, S. R., Steenblock, C., Chrousos, G. P., Schally, A. V., Beuschlein, F., Kline, G., Krone, N. P., Licinio, J., Wong, M. L., Ullmann, E. et al. (2019). Stress-inducible-stem cells: a new view on endocrine, metabolic and mental disease? *Mol. Psychiatry* 24, 2-9. 10.1038/s41380-018-0244-9 30242231 PMC6755998

[JEB246128C13] Bose, R., Moors, M., Tofighi, R., Cascante, A., Hermanson, O. and Ceccatelli, S. (2010). Glucocorticoids induce long-lasting effects in neural stem cells resulting in senescence-related alterations. *Cell Death Dis.* 1, e92. 10.1038/cddis.2010.60 21368868 PMC3032322

[JEB246128C14] Bose, R., Spulber, S., Kilian, P., Heldring, N., Lonnerberg, P., Johnsson, A., Conti, M., Hermanson, O. and Ceccatelli, S. (2015). Tet3 mediates stable glucocorticoid-induced alterations in DNA methylation and Dnmt3a/Dkk1 expression in neural progenitors. *Cell Death Dis.* 6, e1793. 10.1038/cddis.2015.159 26086966 PMC4669838

[JEB246128C15] Boulle, F., VAN Den Hove, D. L. A., Jakob, S. B., Rutten, B. P., Hamon, M., Van Os, J., Lesch, K. P., Lanfumey, L., Steinbusch, H. W. and Kenis, G. (2012). Epigenetic regulation of the BDNF gene: implications for psychiatric disorders. *Mol. Psychiatry* 17, 584-596. 10.1038/mp.2011.107 21894152

[JEB246128C16] Brown, R. W., Diaz, R., Robson, A. C., Kotelevtsev, Y. V., Mullins, J. J., Kaufman, M. H. and Seckl, J. R. (1996). The ontogeny of 11 beta-hydroxysteroid dehydrogenase type 2 and mineralocorticoid receptor gene expression reveal intricate control of glucocorticoid action in development. *Endocrinology* 137, 794-797. 10.1210/endo.137.2.8593833 8593833

[JEB246128C17] Callaghan, B. L. and Tottenham, N. (2016). The stress acceleration hypothesis: effects of early-life adversity on emotion circuits and behavior. *Curr. Opin. Behav. Sci.* 7, 76-81. 10.1016/j.cobeha.2015.11.018 29644262 PMC5890821

[JEB246128C18] Carpenter, T., Grecian, S. M. and Reynolds, R. M. (2017). Sex differences in early-life programming of the hypothalamic-pituitary-adrenal axis in humans suggest increased vulnerability in females: a systematic review. *J. Dev. Orig. Health Dis.* 8, 244-255. 10.1017/S204017441600074X 28103963

[JEB246128C19] Casagrande, S., Stier, A., Monaghan, P., Loveland, J. L., Boner, W., Lupi, S., Trevisi, R. and Hau, M. (2020). Increased glucocorticoid concentrations in early life cause mitochondrial inefficiency and short telomeres. *J. Exp. Biol.* 223, jeb222513. 10.1242/jeb.222513 32532864

[JEB246128C20] Chapman, K., Holmes, M. and Seckl, J. (2013). 11β-hydroxysteroid dehydrogenases: intracellular gate-keepers of tissue glucocorticoid action. *Physiol. Rev.* 93, 1139-1206. 10.1152/physrev.00020.2012 23899562 PMC3962546

[JEB246128C21] Chen, Y., Zhang, J., Tan, H., Li, J. and Yu, Y. (2020). Detrimental effects of hypercortisolism on brain structure and related risk factors. *Sci. Rep.* 10, 12708. 10.1038/s41598-020-68166-0 32728036 PMC7391644

[JEB246128C22] Chin, E. H., Love, O. P., Verspoor, J. J., Williams, T. D., Rowley, K. and Burness, G. (2009). Juveniles exposed to embryonic corticosterone have enhanced flight performance. *Proc. Biol. Sci.* 276, 499-505. 10.1098/rspb.2008.1294 18842541 PMC2664354

[JEB246128C23] Choi, M.-K., Cook, A., Eachus, H., Tochwin, A., Kuntz, S., Kim, S. and Ryu, S. (2023). Lifelong molecular consequences of high Glucocorticoids exposure during development. *bioRxiv, 2023.02.13.528363*.

[JEB246128C24] Costantini, D., Marasco, V. and Møller, A. P. (2011). A meta-analysis of glucocorticoids as modulators of oxidative stress in vertebrates. *J. Comp. Physiol. B* 181, 447-456. 21416253 10.1007/s00360-011-0566-2

[JEB246128C25] Crudo, A., Petropoulos, S., Moisiadis, V. G., Iqbal, M., Kostaki, A., Machnes, Z., Szyf, M. and Matthews, S. G. (2012). Prenatal synthetic glucocorticoid treatment changes DNA methylation states in male organ systems: multigenerational effects. *Endocrinology* 153, 3269-3283. 10.1210/en.2011-2160 22564977 PMC3422463

[JEB246128C26] Crudo, A., Petropoulos, S., Suderman, M., Moisiadis, V. G., Kostaki, A., Hallett, M., Szyf, M. and Matthews, S. G. (2013). Effects of antenatal synthetic glucocorticoid on glucocorticoid receptor binding, DNA methylation, and genome-wide mRNA levels in the fetal male hippocampus. *Endocrinology* 154, 4170-4181. 10.1210/en.2013-1484 24029241

[JEB246128C27] Dantzer, B., Newman, A. E. M., Boonstra, R., Palme, R., Boutin, S., Humphries, M. M. and Mcadam, A. G. (2013). Density triggers maternal hormones that increase adaptive offspring growth in a wild mammal. *Science* 340, 1215-1217. 10.1126/science.1235765 23599265

[JEB246128C28] Dantzer, B., Van Kesteren, F., Westrick, S. E., Boutin, S., Mcadam, A. G., Lane, J. E., Gillespie, R., Majer, A., Haussmann, M. F. and Monaghan, P. (2020). Maternal glucocorticoids promote offspring growth without inducing oxidative stress or shortening telomeres in wild red squirrels. *J. Exp. Biol.* 223, jeb212373. 10.1242/jeb.212373 31796605 PMC10668338

[JEB246128C29] Daskalakis, N. P., Meijer, O. C. and De Kloet, E. R. (2022). Mineralocorticoid receptor and glucocorticoid receptor work alone and together in cell-type-specific manner: implications for resilience prediction and targeted therapy. *Neurobiol. Stress* 18, 100455. 10.1016/j.ynstr.2022.100455 35601687 PMC9118500

[JEB246128C30] Davis, E. P., Sandman, C. A., Buss, C., Wing, D. A. and Head, K. (2013). Fetal glucocorticoid exposure is associated with preadolescent brain development. *Biol. Psychiatry* 74, 647-655. 10.1016/j.biopsych.2013.03.009 23611262 PMC3985475

[JEB246128C31] De Souza, F. J. and Placzek, M. (2021). Conserved roles of Rax/rx3 genes in hypothalamus and pituitary development. *Int. J. Dev. Biol.* 65, 195-205. 10.1387/ijdb.200081fd 32930382

[JEB246128C32] Diamond, D. M., Bennett, M. C., Fleshner, M. and Rose, G. M. (1992). Inverted-U relationship between the level of peripheral corticosterone and the magnitude of hippocampal primed burst potentiation. *Hippocampus* 2, 421-430. 10.1002/hipo.450020409 1308198

[JEB246128C33] Du, J., Wang, Y., Hunter, R., Wei, Y., Blumenthal, R., Falke, C., Khairova, R., Zhou, R., Yuan, P., Machado-Vieira, R. et al. (2009). Dynamic regulation of mitochondrial function by glucocorticoids. *Proc. Natl. Acad. Sci. USA* 106, 3543-3548. 10.1073/pnas.0812671106 19202080 PMC2637276

[JEB246128C34] Dwivedi, Y., Roy, B., Lugli, G., Rizavi, H., Zhang, H. and Smalheiser, N. R. (2015). Chronic corticosterone-mediated dysregulation of microRNA network in prefrontal cortex of rats: relevance to depression pathophysiology. *Transl. Psychiatry* 5, e682-e682. 10.1038/tp.2015.175 26575223 PMC5068767

[JEB246128C35] Eachus, H. and Cunliffe, V. T. (2018). Biological embedding of psychosocial stress over the life course. In *Epigenetics of Aging and Longevity* (Ed. A. Moskalev and A. M. Vaiserman), pp. 254-256. Academic Press.

[JEB246128C36] Eachus, H., Choi, M. K., Tochwin, A., Kaspareit, J., Ho, M. and Ryu, S. (2023a). Elevated glucocorticoid alters the trajectory of hypothalamic development and function. *bioRxiv, 2023.01.27.525966*.

[JEB246128C37] Eachus, H., Oberski, L., Paveley, J., Bacila, I., Ashton, J.-P., Esposito, U., Seifuddin, F., Pirooznia, M., Elhaik, E., Placzek, M. et al. (2023b). Glucocorticoid receptor regulates protein chaperone, circadian clock and affective disorder genes in the zebrafish brain. *Dis. Model. Mech.* 16, dmm050141. 10.1242/dmm.050141 37525888 PMC10565112

[JEB246128C38] Egeland, M., Zunszain, P. A. and Pariante, C. M. (2015). Molecular mechanisms in the regulation of adult neurogenesis during stress. *Nat. Rev. Neurosci.* 16, 189-200. 10.1038/nrn3855 25790864

[JEB246128C39] Encinas, J. M., Michurina, T. V., Peunova, N., Park, J. H., Tordo, J., Peterson, D. A., Fishell, G., Koulakov, A. and Enikolopov, G. (2011). Division-coupled astrocytic differentiation and age-related depletion of neural stem cells in the adult hippocampus. *Cell Stem Cell* 8, 566-579. 10.1016/j.stem.2011.03.010 21549330 PMC3286186

[JEB246128C40] Fitzsimons, C. P., Herbert, J., Schouten, M., Meijer, O. C., Lucassen, P. J. and Lightman, S. (2016). Circadian and ultradian glucocorticoid rhythmicity: implications for the effects of glucocorticoids on neural stem cells and adult hippocampal neurogenesis. *Front. Neuroendocrinol.* 41, 44-58. 10.1016/j.yfrne.2016.05.001 27234350

[JEB246128C41] Goodman, T. and Hajihosseini, M. K. (2015). Hypothalamic tanycytes-masters and servants of metabolic, neuroendocrine, and neurogenic functions. *Front. Neurosci.* 9, 387. 10.3389/fnins.2015.00387 26578855 PMC4624852

[JEB246128C42] Gray, J. D., Kogan, J. F., Marrocco, J. and Mcewen, B. S. (2017). Genomic and epigenomic mechanisms of glucocorticoids in the brain. *Nat. Rev. Endocrinol.* 13, 661-673. 10.1038/nrendo.2017.97 28862266

[JEB246128C43] Groeneweg, F. L., Karst, H., De Kloet, E. R. and Joëls, M. (2012). Mineralocorticoid and glucocorticoid receptors at the neuronal membrane, regulators of nongenomic corticosteroid signalling. *Mol. Cell. Endocrinol.* 350, 299-309. 10.1016/j.mce.2011.06.020 21736918

[JEB246128C44] Haase, C. G., Long, A. K. and Gillooly, J. F. (2016). Energetics of stress: linking plasma cortisol levels to metabolic rate in mammals. *Biol. Lett.* 12, 20150867. 10.1098/rsbl.2015.0867 26740562 PMC4785924

[JEB246128C45] Haussmann, M. F., Longenecker, A. S., Marchetto, N. M., Juliano, S. A. and Bowden, R. M. (2012). Embryonic exposure to corticosterone modifies the juvenile stress response, oxidative stress and telomere length. *Proc. Biol. Sci.* 279, 1447-1456. 10.1098/rspb.2011.1913 22072607 PMC3282378

[JEB246128C46] Hayashi-Takagi, A., Yagishita, S., Nakamura, M., Shirai, F., Wu, Y. I., Loshbaugh, A. L., Kuhlman, B., Hahn, K. M. and Kasai, H. (2015). Labelling and optical erasure of synaptic memory traces in the motor cortex. *Nature* 525, 333-338. 10.1038/nature15257 26352471 PMC4634641

[JEB246128C47] Hill, E. E., Zack, E., Battaglini, C., Viru, M., Viru, A. and Hackney, A. C. (2008). Exercise and circulating cortisol levels: the intensity threshold effect. *J. Endocrinol. Invest.* 31, 587-591. 10.1007/BF03345606 18787373

[JEB246128C48] Holmes, M. C., Sangra, M., French, K. L., Whittle, I. R., Paterson, J., Mullins, J. J. and Seckl, J. R. (2006). 11β-Hydroxysteroid dehydrogenase type 2 protects the neonatal cerebellum from deleterious effects of glucocorticoids. *Neuroscience* 137, 865-873. 10.1016/j.neuroscience.2005.09.037 16289840 PMC6443040

[JEB246128C49] Horvath, S. (2013). DNA methylation age of human tissues and cell types. *Genome Biol.* 14, 3156. 10.1186/gb-2013-14-10-r115 PMC4015143 24138928

[JEB246128C50] Inoue, R., Abdou, K., Hayashi-Tanaka, A., Muramatsu, S. I., Mino, K., Inokuchi, K. and Mori, H. (2018). Glucocorticoid receptor-mediated amygdalar metaplasticity underlies adaptive modulation of fear memory by stress. *Elife* 7, e34135. 10.7554/eLife.34135 29941090 PMC6019067

[JEB246128C51] Ito, K., Barnes, P. J. and Adcock, I. M. (2000). Glucocorticoid receptor recruitment of histone deacetylase 2 inhibits interleukin-1beta-induced histone H4 acetylation on lysines 8 and 12. *Mol. Cell. Biol.* 20, 6891-6903. 10.1128/MCB.20.18.6891-6903.2000 10958685 PMC88765

[JEB246128C52] Jawahar, M. C., Murgatroyd, C., Harrison, E. L. and Baune, B. T. (2015). Epigenetic alterations following early postnatal stress: a review on novel aetiological mechanisms of common psychiatric disorders. *Clin. Epigenetics* 7, 122. 10.1186/s13148-015-0156-3 26583053 PMC4650349

[JEB246128C53] Joëls, M. (2006). Corticosteroid effects in the brain: U-shape it. *Trends Pharmacol. Sci.* 27, 244-250. 10.1016/j.tips.2006.03.007 16584791

[JEB246128C54] Kanagawa, T., Tomimatsu, T., Hayashi, S., Shioji, M., Fukuda, H., Shimoya, K. and Murata, Y. (2006). The effects of repeated corticosteroid administration on the neurogenesis in the neonatal rat. *Am. J. Obstet. Gynecol.* 194, 231-238. 10.1016/j.ajog.2005.06.015 16389037

[JEB246128C55] Karst, H., Berger, S., Erdmann, G., Schutz, G. and Joels, M. (2010). Metaplasticity of amygdalar responses to the stress hormone corticosterone. *Proc. Natl. Acad. Sci. USA* 107, 14449-14454. 10.1073/pnas.0914381107 20663957 PMC2922581

[JEB246128C56] Kirby, E. D., Muroy, S. E., Sun, W. G., Covarrubias, D., Leong, M. J., Barchas, L. A. and Kaufer, D. (2013). Acute stress enhances adult rat hippocampal neurogenesis and activation of newborn neurons via secreted astrocytic FGF2. *Elife* 2, e00362. 10.7554/eLife.00362 23599891 PMC3628086

[JEB246128C57] Klengel, T., Mehta, D., Anacker, C., Rex-Haffner, M., Pruessner, J. C., Pariante, C. M., Pace, T. W. W., Mercer, K. B., Mayberg, H. S., Bradley, B. et al. (2013). Allele-specific FKBP5 DNA demethylation mediates gene-childhood trauma interactions. *Nat. Neurosci.* 16, 33-41. 10.1038/nn.3275 23201972 PMC4136922

[JEB246128C58] Kõks, S., Dogan, S., Tuna, B. G., González-Navarro, H., Potter, P. and Vandenbroucke, R. E. (2016). Mouse models of ageing and their relevance to disease. *Mech. Ageing Dev.* 160, 41-53. 10.1016/j.mad.2016.10.001 27717883

[JEB246128C59] Konefal, S., Elliot, M. and Crespi, B. (2013). The adaptive significance of adult neurogenesis: an integrative approach. *Front. Neuroanat.* 7, 21. 10.3389/fnana.2013.00021 23882188 PMC3712125

[JEB246128C60] Koning, A., Buurstede, J. C., Van Weert, L. and Meijer, O. C. (2019). Glucocorticoid and mineralocorticoid receptors in the brain: a transcriptional perspective. *J. Endocr. Soc.* 3, 1917-1930. 10.1210/js.2019-00158 31598572 PMC6777400

[JEB246128C61] Korosi, A., Naninck, E. F., Oomen, C. A., Schouten, M., Krugers, H., Fitzsimons, C. and Lucassen, P. J. (2012). Early-life stress mediated modulation of adult neurogenesis and behavior. *Behav. Brain Res.* 227, 400-409. 10.1016/j.bbr.2011.07.037 21821065

[JEB246128C62] Kumari, R. and Jat, P. (2021). Mechanisms of cellular senescence: cell cycle arrest and senescence associated secretory phenotype. *Front. Cell Dev. Biol.* 9, 645593. 10.3389/fcell.2021.645593 33855023 PMC8039141

[JEB246128C63] Lee, R. S., Tamashiro, K. L., Yang, X., Purcell, R. H., Harvey, A., Willour, V. L., Huo, Y., Rongione, M., Wand, G. S. and Potash, J. B. (2010). Chronic corticosterone exposure increases expression and decreases deoxyribonucleic acid methylation of Fkbp5 in mice. *Endocrinology* 151, 4332-4343. 10.1210/en.2010-0225 20668026 PMC2940504

[JEB246128C64] Lee, D. A., Bedont, J. L., Pak, T., Wang, H., Song, J., Miranda-Angulo, A., Takiar, V., Charubhumi, V., Balordi, F., Takebayashi, H. et al. (2012). Tanycytes of the hypothalamic median eminence form a diet-responsive neurogenic niche. *Nat. Neurosci.* 15, 700-702. 10.1038/nn.3079 22446882 PMC3380241

[JEB246128C65] Lee, W.-S., Monaghan, P. and Metcalfe, N. B. (2013). Experimental demonstration of the growth rate–lifespan trade-off. *Proc. R. Soc. B* 280, 20122370. 10.1098/rspb.2012.2370 PMC3574304 23235704

[JEB246128C66] Lehmann, M. L., Brachman, R. A., Martinowich, K., Schloesser, R. J. and Herkenham, M. (2013). Glucocorticoids orchestrate divergent effects on mood through adult neurogenesis. *J. Neurosci.* 33, 2961-2972. 10.1523/JNEUROSCI.3878-12.2013 23407954 PMC3711562

[JEB246128C67] Liston, C. and Gan, W. B. (2011). Glucocorticoids are critical regulators of dendritic spine development and plasticity in vivo. *Proc. Natl. Acad. Sci. USA* 108, 16074-16079. 10.1073/pnas.1110444108 21911374 PMC3179117

[JEB246128C68] Liston, C., Cichon, J. M., Jeanneteau, F., Jia, Z., Chao, M. V. and Gan, W. B. (2013). Circadian glucocorticoid oscillations promote learning-dependent synapse formation and maintenance. *Nat. Neurosci.* 16, 698-705. 10.1038/nn.3387 23624512 PMC3896394

[JEB246128C69] Liu, P. Z. and Nusslock, R. (2018a). Exercise-mediated neurogenesis in the hippocampus via BDNF. *Front. Neurosci.* 12, 52. 10.3389/fnins.2018.00052 29467613 PMC5808288

[JEB246128C70] Liu, P. Z. and Nusslock, R. (2018b). How stress gets under the skin: early life adversity and glucocorticoid receptor epigenetic regulation. *Curr. Genomics* 19, 653-664. 10.2174/1389202919666171228164350 30532645 PMC6225447

[JEB246128C71] Lupien, S. J., Mcewen, B. S., Gunnar, M. R. and Heim, C. (2009). Effects of stress throughout the lifespan on the brain, behaviour and cognition. *Nat. Rev. Neurosci.* 10, 434-445. 10.1038/nrn2639 19401723

[JEB246128C72] Magalhães, R., Gonçalves, N., Sousa, R., Coelho, A., Soares-Cunha, C., Moreira, P., Marques, P., Tuulari, J. J., Scheinin, N. M., Karlsson, L. et al. (2023). Impact of prenatal synthetic glucocorticoid exposure on the adolescent brain. *BioRxiv*. 10.1101/2023.04.14.536872

[JEB246128C73] Matamales, M., Skrbis, Z., Hatch, R. J., Balleine, B. W., Götz, J. and Bertran-Gonzalez, J. (2016). Aging-related dysfunction of striatal cholinergic interneurons produces conflict in action selection. *Neuron* 90, 362-373. 10.1016/j.neuron.2016.03.006 27100198

[JEB246128C74] McEwen, B. S. and Gianaros, P. J. (2011). Stress- and allostasis-induced brain plasticity. *Annu. Rev. Med.* 62, 431-445. 10.1146/annurev-med-052209-100430 20707675 PMC4251716

[JEB246128C75] McEwen, B. S. and Liston, C. (2017). Mediators of glucocorticoid-regulated adaptive plasticity. In *The Oxford Handbook of Developmental Neural Plasticity* (ed. M. V. Chao). Oxford Academic.

[JEB246128C76] McEwen, B. S. and Wingfield, J. C. (2003). The concept of allostasis in biology and biomedicine. *Horm. Behav.* 43, 2-15. 10.1016/S0018-506X(02)00024-7 12614627

[JEB246128C77] Metcalfe, N. B. and Olsson, M. (2022). How telomere dynamics are influenced by the balance between mitochondrial efficiency, reactive oxygen species production and DNA damage. *Mol. Ecol.* 31, 6040-6052. 10.1111/mec.16150 34435398

[JEB246128C78] Mifsud, K. R. and Reul, J. M. H. M. (2018). Mineralocorticoid and glucocorticoid receptor-mediated control of genomic responses to stress in the brain. *Stress* 21, 389-402. 10.1080/10253890.2018.1456526 29614900

[JEB246128C79] Mifsud, K. R., Kennedy, C. L. M., Salatino, S., Sharma, E., Price, E. M., Haque, S. N., Gialeli, A., Goss, H. M., Panchenko, P. E., Broxholme, J. et al. (2021). Distinct regulation of hippocampal neuroplasticity and ciliary genes by corticosteroid receptors. *Nat. Commun.* 12, 4737. 10.1038/s41467-021-24967-z 34362910 PMC8346558

[JEB246128C80] Miranda, J. M., Cruz, E., Bessières, B. and Alberini, C. M. (2022). Hippocampal parvalbumin interneurons play a critical role in memory development. *Cell Rep.* 41, 111643. 10.1016/j.celrep.2022.111643 36384113 PMC9737056

[JEB246128C81] Mirescu, C., Peters, J. D. and Gould, E. (2004). Early life experience alters response of adult neurogenesis to stress. *Nat. Neurosci.* 7, 841-846. 10.1038/nn1290 15273691

[JEB246128C82] Moisiadis, V. G. and Matthews, S. G. (2014). Glucocorticoids and fetal programming part 1: outcomes. *Nat. Rev. Endocrinol.* 10, 391-402. 10.1038/nrendo.2014.73 24863382

[JEB246128C83] Monaghan, P., Heidinger, B. J., D'alba, L., Evans, N. P. and Spencer, K. A. (2012). For better or worse: reduced adult lifespan following early-life stress is transmitted to breeding partners. *Proc. Biol. Sci.* 279, 709-714. 21849320 10.1098/rspb.2011.1291 PMC3248736

[JEB246128C84] Mourtzi, N., Sertedaki, A. and Charmandari, E. (2021). Glucocorticoid signaling and epigenetic alterations in stress-related disorders. *Int. J. Mol. Sci.* 22, 5964. 10.3390/ijms22115964 34073101 PMC8198182

[JEB246128C85] Muthu, V., Eachus, H., Ellis, P., Brown, S. and Placzek, M. (2016). Rx3 and Shh direct anisotropic growth and specification in the zebrafish tuberal/anterior hypothalamus. *Development* 143, 2651-2663. 27317806 10.1242/dev.138305 PMC4958342

[JEB246128C86] Noorlander, C. W., Tijsseling, D., Hessel, E. V., De Vries, W. B., Derks, J. B., Visser, G. H. and De Graan, P. N. (2014). Antenatal glucocorticoid treatment affects hippocampal development in mice. *PLoS One* 9, e85671. 10.1371/journal.pone.0085671 24465645 PMC3899077

[JEB246128C87] Okano, M., Bell, D. W., Haber, D. A. and Li, E. (1999). DNA methyltransferases Dnmt3a and Dnmt3b are essential for de novo methylation and mammalian development. *Cell* 99, 247-257. 10.1016/S0092-8674(00)81656-6 10555141

[JEB246128C88] Panettieri, R. A., Schaafsma, D., Amrani, Y., Koziol-White, C., Ostrom, R. and Tliba, O. (2019). Non-genomic effects of glucocorticoids: an updated view. *Trends Pharmacol. Sci.* 40, 38-49. 10.1016/j.tips.2018.11.002 30497693 PMC7106476

[JEB246128C89] Parel, S. T. and Peña, C. J. (2022). Genome-wide signatures of early-life stress: influence of sex. *Biol. Psychiatry* 91, 36-42. 10.1016/j.biopsych.2020.12.010 33602500 PMC8791071

[JEB246128C90] Pariante, C. M. and Lightman, S. L. (2008). The HPA axis in major depression: classical theories and new developments. *Trends Neurosci.* 31, 464-468. 10.1016/j.tins.2008.06.006 18675469

[JEB246128C91] Pettigrew, C. and Martin, R. C. (2014). Cognitive declines in healthy aging: evidence from multiple aspects of interference resolution. *Psychol. Aging* 29, 187. 10.1037/a0036085 24955989

[JEB246128C92] Pietropaolo, S. and Marsicano, G. (2022). The role of the endocannabinoid system as a therapeutic target for autism spectrum disorder: lessons from behavioral studies on mouse models. *Neurosci. Biobehav. Rev.* 132, 664-678. 10.1016/j.neubiorev.2021.11.031 34813825

[JEB246128C93] Prekovic, S., Schuurman, K., Mayayo-Peralta, I., Manjón, A. G., Buijs, M., Yavuz, S., Wellenstein, M. D., Barrera, A., Monkhorst, K., Huber, A. et al. (2021). Glucocorticoid receptor triggers a reversible drug-tolerant dormancy state with acquired therapeutic vulnerabilities in lung cancer. *Nat. Commun.* 12, 4360. 10.1038/s41467-021-24537-3 34272384 PMC8285479

[JEB246128C94] Provençal, N. and Binder, E. B. (2015). The effects of early life stress on the epigenome: from the womb to adulthood and even before. *Exp. Neurol.* 268, 10-20. 10.1016/j.expneurol.2014.09.001 25218020

[JEB246128C95] Provençal, N., Arloth, J., Cattaneo, A., Anacker, C., Cattane, N., Wiechmann, T., Röh, S., Ködel, M., Klengel, T., Czamara, D. et al. (2020). Glucocorticoid exposure during hippocampal neurogenesis primes future stress response by inducing changes in DNA methylation. *Proc. Natl. Acad. Sci. USA* 117, 23280-23285. 10.1073/pnas.1820842116 31399550 PMC7519233

[JEB246128C96] Quirarte, G. L., Roozendaal, B. and Mcgaugh, J. L. (1997). Glucocorticoid enhancement of memory storage involves noradrenergic activation in the basolateralamygdala. *Proc. Natl. Acad. Sci. USA* 94, 14048-14053. 10.1073/pnas.94.25.14048 9391150 PMC28430

[JEB246128C97] Rahman, M. F. and Mcgowan, P. O. (2022). Cell-type-specific epigenetic effects of early life stress on the brain. *Transl. Psychiatry* 12, 326. 10.1038/s41398-022-02076-9 35948532 PMC9365848

[JEB246128C98] Rahner-Welsch, S., Frölich, L., Stoll, S. and Hoyer, S. (1995). Decline and preservation of reversal learning abilities and acquisition in the course of senescence. *Neurosci. Lett.* 194, 121-123. 10.1016/0304-3940(95)11712-6 7478192

[JEB246128C99] Reul, J. M. and De Kloet, E. R. (1985). Two receptor systems for corticosterone in rat brain: microdistribution and differential occupation. *Endocrinology* 117, 2505-2511. 10.1210/endo-117-6-2505 2998738

[JEB246128C100] Reul, J. M., VAN Den Bosch, F. R. and De Kloet, E. R. (1987). Relative occupation of type-I and type-II corticosteroid receptors in rat brain following stress and dexamethasone treatment: functional implications. *J. Endocrinol.* 115, 459-467. 10.1677/joe.0.1150459 3443807

[JEB246128C101] Reyes-Contreras, M. and Taborsky, B. (2022). Stress axis programming generates long-term effects on cognitive abilities in a cooperative breeder. *Proc. Biol. Sci.* 289, 20220117. 10.1098/rspb.2022.0117 35582802 PMC9114936

[JEB246128C102] Reyes-Contreras, M., Glauser, G., Rennison, D. J. and Taborsky, B. (2019). Early-life manipulation of cortisol and its receptor alters stress axis programming and social competence. *Philos. Trans. R. Soc. Lond. B Biol. Sci.* 374, 20180119. 10.1098/rstb.2018.0119 30966879 PMC6460083

[JEB246128C103] Ryu, S. and De Marco, R. J. (2017). Performance on innate behaviour during early development as a function of stress level. *Sci. Rep.* 7, 7840. 10.1038/s41598-017-08400-4 28798473 PMC5552790

[JEB246128C104] Saaltink, D. J. and Vreugdenhil, E. (2014). Stress, glucocorticoid receptors, and adult neurogenesis: a balance between excitation and inhibition? *Cell. Mol. Life Sci.* 71, 2499-2515. 10.1007/s00018-014-1568-5 24522255 PMC4055840

[JEB246128C105] Salazar-Roa, M. and Malumbres, M. (2017). Fueling the cell division cycle. *Trends Cell Biol.* 27, 69-81. 10.1016/j.tcb.2016.08.009 27746095

[JEB246128C106] Sasaki, A., Eng, M. E., Lee, A. H., Kostaki, A. and Matthews, S. G. (2021). DNA methylome signatures of prenatal exposure to synthetic glucocorticoids in hippocampus and peripheral whole blood of female guinea pigs in early life. *Transl. Psychiatry* 11, 63. 10.1038/s41398-020-01186-6 33462183 PMC7813870

[JEB246128C107] Sawamura, T., Klengel, T., Armario, A., Jovanovic, T., Norrholm, S. D., Ressler, K. J. and Andero, R. (2016). Dexamethasone treatment leads to enhanced fear extinction and dynamic Fkbp5 regulation in amygdala. *Neuropsychopharmacology* 41, 832-846. 10.1038/npp.2015.210 26174596 PMC4707829

[JEB246128C108] Schmidt, M. V. (2019). Stress-hyporesponsive period. In *Stress: Physiology, Biochemistry, and Pathology* (ed. G. Fink), pp. 49-56. Academic Press.

[JEB246128C109] Schouten, M., Bielefeld, P., Garcia-Corzo, L., Passchier, E. M. J., Gradari, S., Jungenitz, T., Pons-Espinal, M., Gebara, E., Martín-Suárez, S., Lucassen, P. J. et al. (2020). Circadian glucocorticoid oscillations preserve a population of adult hippocampal neural stem cells in the aging brain. *Mol. Psychiatry* 25, 1382-1405. 10.1038/s41380-019-0440-2 31222184 PMC7303016

[JEB246128C110] Seifuddin, F., Wand, G., Cox, O., Pirooznia, M., Moody, L., Yang, X., Tai, J., Boersma, G., Tamashiro, K., Zandi, P. et al. (2017). Genome-wide Methyl-Seq analysis of blood-brain targets of glucocorticoid exposure. *Epigenetics* 12, 637-652. 10.1080/15592294.2017.1334025 28557603 PMC5687336

[JEB246128C111] Setiawan, E., Jackson, M. F., Macdonald, J. F. and Matthews, S. G. (2007). Effects of repeated prenatal glucocorticoid exposure on long-term potentiation in the juvenile guinea-pig hippocampus. *J. Physiol.* 581, 1033-1042. 10.1113/jphysiol.2006.127381 17412773 PMC2170854

[JEB246128C112] Singewald, N. and Holmes, A. (2019). Rodent models of impaired fear extinction. *Psychopharmacology* 236, 21-32. 10.1007/s00213-018-5054-x 30377749 PMC6373188

[JEB246128C113] So, J. H., Huang, C., Ge, M., Cai, G., Zhang, L., Lu, Y. and Mu, Y. (2017). Intense exercise promotes adult hippocampal neurogenesis but not spatial discrimination. *Front. Cell. Neurosci.* 11, 13. 10.3389/fncel.2017.00013 28197080 PMC5281566

[JEB246128C114] Starkman, M. N., Giordani, B., Gebarski, S. S., Berent, S., Schork, M. A. and Schteingart, D. E. (1999). Decrease in cortisol reverses human hippocampal atrophy following treatment of Cushing's disease. *Biol. Psychiatry* 46, 1595-1602. 10.1016/S0006-3223(99)00203-6 10624540

[JEB246128C115] Sundberg, M., Savola, S., Hienola, A., Korhonen, L. and Lindholm, D. (2006). Glucocorticoid hormones decrease proliferation of embryonic neural stem cells through ubiquitin-mediated degradation of cyclin D1. *J. Neurosci.* 26, 5402-5410. 10.1523/JNEUROSCI.4906-05.2006 16707792 PMC6675314

[JEB246128C116] Surget, A. and Belzung, C. (2022). Adult hippocampal neurogenesis shapes adaptation and improves stress response: a mechanistic and integrative perspective. *Mol. Psychiatry* 27, 403-421. 10.1038/s41380-021-01136-8 33990771 PMC8960391

[JEB246128C117] Teissier, A., Le Magueresse, C., Olusakin, J., Andrade Da Costa, B. L. S., De Stasi, A. M., Bacci, A., Imamura Kawasawa, Y., Vaidya, V. A. and Gaspar, P. (2020). Early-life stress impairs postnatal oligodendrogenesis and adult emotional behaviour through activity-dependent mechanisms. *Mol. Psychiatry* 25, 1159-1174. 10.1038/s41380-019-0493-2 31439936 PMC7244403

[JEB246128C118] Timmermans, S., Souffriau, J. and Libert, C. (2019). A general introduction to glucocorticoid biology. *Front. Immunol.* 10, 1545. 10.3389/fimmu.2019.01545 31333672 PMC6621919

[JEB246128C119] Tooley, U. A., Bassett, D. S. and Mackey, A. P. (2021). Environmental influences on the pace of brain development. *Nat. Rev. Neurosci.* 22, 372-384. 10.1038/s41583-021-00457-5 33911229 PMC8081006

[JEB246128C120] Torres-Berrío, A., Issler, O., Parise, E. M. and Nestler, E. J. (2019). Unraveling the epigenetic landscape of depression: focus on early life stress . *Dialogues Clin. Neurosci.* 21, 341-357. 10.31887/DCNS.2019.21.4/enestler 31949402 PMC6952747

[JEB246128C121] Tovo-Neto, A., Martinez, E. R. M., Melo, A. G., Doretto, L. B., Butzge, A. J., Rodrigues, M. S., Nakajima, R. T., Habibi, H. R. and Nobrega, R. H. (2020). Cortisol directly stimulates spermatogonial differentiation, meiosis, and spermiogenesis in zebrafish (*Danio rerio*) testicular explants. *Biomolecules* 10, 429. 10.3390/biom10030429 32164184 PMC7175196

[JEB246128C122] Trinchero, M. F., Herrero, M. and Schinder, A. F. (2019). Rejuvenating the brain with chronic exercise through adult neurogenesis. *Front. Neurosci.* 13, 1000. 10.3389/fnins.2019.01000 31619959 PMC6759473

[JEB246128C123] Van Der Meulen, M., Amaya, J. M., Dekkers, O. M. and Meijer, O. C. (2022). Association between use of systemic and inhaled glucocorticoids and changes in brain volume and white matter microstructure: a cross-sectional study using data from the UK Biobank. *BMJ Open* 12, e062446. 10.1136/bmjopen-2022-062446 PMC9438037 36041764

[JEB246128C124] Vockley, C. M., D'ippolito, A. M., Mcdowell, I. C., Majoros, W. H., Safi, A., Song, L., Crawford, G. E. and Reddy, T. E. (2016). Direct GR binding sites potentiate clusters of TF binding across the human genome. *Cell* 166, 1269-1281.e19. 10.1016/j.cell.2016.07.049 27565349 PMC5046229

[JEB246128C125] Wiechmann, T., Röh, S., Sauer, S., Czamara, D., Arloth, J., Ködel, M., Beintner, M., Knop, L., Menke, A., Binder, E. B. et al. (2019). Identification of dynamic glucocorticoid-induced methylation changes at the FKBP5 locus. *Clin. Epigenetics* 11, 83. 10.1186/s13148-019-0682-5 31122292 PMC6533766

[JEB246128C126] Wyrwoll, C. S., Holmes, M. C. and Seckl, J. R. (2011). 11β-hydroxysteroid dehydrogenases and the brain: from zero to hero, a decade of progress. *Front. Neuroendocrinol.* 32, 265-286. 10.1016/j.yfrne.2010.12.001 21144857 PMC3149101

[JEB246128C127] Wyrwoll, C., Keith, M., Noble, J., Stevenson, P. L., Bombail, V., Crombie, S., Evans, L. C., Bailey, M. A., Wood, E., Seckl, J. R. et al. (2015). Fetal brain 11β-hydroxysteroid dehydrogenase type 2 selectively determines programming of adult depressive-like behaviors and cognitive function, but not anxiety behaviors in male mice. *Psychoneuroendocrinology* 59, 59-70. 10.1016/j.psyneuen.2015.05.003 26036451 PMC4510145

[JEB246128C128] Yang, F., Zhang, F., Ji, X., Jiang, X., Xue, M., Yu, H., Hu, X. and Bao, Z. (2020). Secretory galectin-3 induced by glucocorticoid stress triggers stemness exhaustion of hepatic progenitor cells. *J. Biol. Chem.* 295, 16852-16862. 10.1074/jbc.RA120.012974 32989051 PMC7864077

[JEB246128C129] Youssef, M., Atsak, P., Cardenas, J., Kosmidis, S., Leonardo, E. D. and Dranovsky, A. (2019). Early life stress delays hippocampal development and diminishes the adult stem cell pool in mice. *Sci. Rep.* 9, 4120. 10.1038/s41598-019-40868-0 30858462 PMC6412041

[JEB246128C130] Zannas, A. S. and Chrousos, G. P. (2017). Epigenetic programming by stress and glucocorticoids along the human lifespan. *Mol. Psychiatry* 22, 640-646. 10.1038/mp.2017.35 28289275

[JEB246128C131] Zannas, A. S., Arloth, J., Carrillo-Roa, T., Iurato, S., Roh, S., Ressler, K. J., Nemeroff, C. B., Smith, A. K., Bradley, B., Heim, C. et al. (2015). Lifetime stress accelerates epigenetic aging in an urban, African American cohort: relevance of glucocorticoid signaling. *Genome Biol.* 16, 266. 10.1186/s13059-015-0828-5 26673150 PMC4699359

[JEB246128C132] Zannas, A. S., Wiechmann, T., Gassen, N. C. and Binder, E. B. (2016). Gene– stress–epigenetic regulation of FKBP5: clinical and translational implications. *Neuropsychopharmacology* 41, 261-274. 10.1038/npp.2015.235 26250598 PMC4677131

[JEB246128C133] Zannas, A. S., Jia, M., Hafner, K., Baumert, J., Wiechmann, T., Pape, J. C., Arloth, J., Ködel, M., Martinelli, S., Roitman, M. et al. (2019). Epigenetic upregulation of FKBP5 by aging and stress contributes to NF-κB-driven inflammation and cardiovascular risk. *Proc. Natl. Acad. Sci. USA* 116, 11370-11379. 10.1073/pnas.1816847116 31113877 PMC6561294

[JEB246128C134] Zheng, Y., Fan, W., Zhang, X. and Dong, E. (2016). Gestational stress induces depressive-like and anxiety-like phenotypes through epigenetic regulation of BDNF expression in offspring hippocampus. *Epigenetics* 11, 150-162. 10.1080/15592294.2016.1146850 26890656 PMC4846107

[JEB246128C135] Ziv, L., Muto, A., Schoonheim, P. J., Meijsing, S. H., Strasser, D., Ingraham, H. A., Schaaf, M. J. M., Yamamoto, K. R. and Baier, H. (2013). An affective disorder in zebrafish with mutation of the glucocorticoid receptor. *Mol. Psychiatry* 18, 681-691. 10.1038/mp.2012.64 22641177 PMC4065652

